# A Genomic Signature for Genotyping Mycobacterium tuberculosis

**DOI:** 10.6026/97320630013224

**Published:** 2017-07-31

**Authors:** David Tarazona, Luis Jaramillo, Victor Borda, Kelly Levano, Marco Galarza, Heinner Guio

**Affiliations:** 1 Laboratorio de Biotecnología y Biología Molecular, Centro Nacional de Salud Pública, Instituto Nacional de Salud, Lima, Perú.

**Keywords:** Genomic signature, Genotyping, Mycobacterium tuberculosis

## Abstract

Mycobacterium tuberculosis (MTB), the causative agent of tuberculosis (TB), has a vast diversity of genotypes including Beijing, CAS, EAI, Haarlem, LAM, X, Ural, T, AFRI1 and AFRI2. However, genotyping can be expensive, time consuming and in some cases, results may vary depending on methodology used. Here, we proposed a new set of 10 SNPs using a total of 249 MTB genomes, and selected by first the inclusion/ exclusion (IE) criteria using spoligotyping and phylogenies, followed by the selection of the nonsynonymous SNPs present in the most conserved cluster of orthologous groups (COG) of each genotype of MTB. Genotype assignment of the new set of 10 SNPs was validated using an additional of 34 MTB genomes and results showed 100% correlation with their known genotypes. Our set of 10 SNPs have not been previously reported and cover the MTB genotypes that are prevalent worldwide. This set of SNPs could be used for molecular epidemiology with drug resistant markers.

## Background

Tuberculosis (TB), responsible for approximately 1.4 million deaths annually, represents one of the main challenges for public health. The decoding of the M. tuberculosis (MTB) genome [1] has accelerated the advances in its genetic diversity and TB diagnosis. MTB families like Beijing, Latin American (LAM), Haarlem, Central Asian (CAS), T, East-African-Indian (EAI), Euro- American (X-type) and AFRI have been established via MIRUVNTR and spoligotyping [2,3, 4,5]. These techniques are based on the number of tandem intergenic repeats in the genome of MTB [6] and the amplification by PCR of clustered regulatory short palindromic repeats (CRISPR) [7], respectively. The development of genotyping methodologies has allowed: (1) comparison of strains circulating in different populations, (2) characterization of outbreaks, (3) distinction between recent and old transmissions, (4) detection of cross-contamination in laboratories, (5) detection of re-infection or relapse and (6) identification of populations with high risk of transmission allowing subsequent implementation of appropriate control strategy [8,9, 10]. At present, there are regions with higher frequency of TB infection and a particular MTB genotype [11]. However, countries with a heterogeneous population, such as the United States [12], Peru [13], Israel [14], European countries [15, 16] and even China [17], have reported a greater diversity of MTB families which complicate their distribution and evolution. This is where genotyping becomes a challenge with the current methodologies MIRU-VNTR and spoligotyping, which lack sufficient discriminatory power to differentiate between families [18]. Whole genome sequencing (WGS) has been shown to give superior resolution to that of MIRU-VNTR and spoligotyping providing all possible genomic targets, information on drug resistance, genome evolution and virulence determinants [19]. However, massive sequencing costs are still expensive in countries with high TB incidence. The best approach will be to analyze a specific set of SNPs associated to a determined genotype. Recent studies have proposed sets of SNPs, one using 45 SNPs from 04 genomic sequences [20] and another using 62 SNPs from 1601 genomes [21]. However, these two studies have been based on phylogenies. In this study, we proposed a new set of 10 SNPs using a total of 249 MTB genomes selected first by the inclusion/ exclusion (IE) criteria using spoligotyping and phylogenies, followed by the selection of the nonsynonymous SNPs present in the most conserved COG (cluster of orthologous groups) of each genotype (Beijing, CAS, EAI, Haarlem, LAM, X, Ural, T, AFRI1 and AFRI2) of MTB. The addition of spoligotyping provides higher informative results on the phylogeographic distribution of MTB׳s genotypic diversity [22].

## Methodology

### Genome sequences selection

A total of 249 MTB genomic sequences were used in this study: 125 of the genomic sequences had known genotypes determined by the gold standard method (spoligotyping), including Beijing (lineage 2), CAS (lineage 3), EAI (lineage 1 y 3), Haarlem (lineage 4), LAM (lineage 4), X genotypes (lineage 4), Ural (lineage 4), T (lineage 4), AFRI1 (lineage 5) and AFRI2 (lineage 6), based on publications and public databases; 76 of the genomic sequences had known genotypes determined by phylogenetic analysis including lineage 1 (EAI), lineage 2 (Beijing), lineage 3 (CAS and EAI), lineage 4 (Haarlem, LAM, T, X), lineage 5 (AFRI1) and lineage 6 (AFR2); and 48 of the genomic sequences has unknown genotypes. These genomic sequences were obtained from public domains of the international database from the National Institute of Health (NIH) (ftp://ftp.ncbi.nlm.nih.gov/genomes/), European Nucleotide Archive (ENA; http://www.ebi.ac.uk/ena/) and Kyoto Encyclopedia of Genes and Genomes (KEGG) (http://www.genome.jp/en/gn_ftp.html). The MTB sequences analyzed were from 35 countries (USA, Canada, China, Colombia, El Salvador, Ethiopia, Gambia, Germany, Ghana, Guatemala, India, Italia, Japan, Kazakhstan, Malaysia, Mexico, Mongolia, Nepal, Netherlands, Nicaragua, Panama, Peru, Philippines, Puerto Rico, Russia, Sierra Leone, South Africa, Sweden, Tanzania, Iran, Thailand, Uganda, United Kingdom, Uruguay and Vietnam) allowing us to obtain a global diversity and MTB representativeness. It is a retrospective study so ethical approval was not required.

Additionally, another 34 MTB genomic sequences with known genotypes: Beijing (n=12), LAM (n=07), X (n=06), CAS (n=04), Haarlem (n=04) and EAI (n=01) were used for genotype confirmation using our new proposed set of 10 SNPs. Analyzed data were mapped to the H37Rv reference genome (number accession: NC_000962.3) using BWA [23]. SAMtools [24], kSNP [25] and ParSNP were used to identify SNPs. These programs exclude the analysis of SNPs in non-sequenced regions.

### Phylogenetic analysis of genomic sequences

Phylogenetic assays were performed on 173 MTB genomes (125 with genotypes determined by spoligotyping and 48 with unknown genotypes) using two different programs of alignment free: kSNP [26] and ParSNP [27]. Each program uses different algorithms to select SNPs, constructs the Maximum likelihood phylogenies and compares topologies. kSNP identifies SNPs based on k-mers analysis without using a reference genome. This program requires a k-value for the mer size, which is calculated by Kchooser tool. Also, in order to count the k-mers in the genomes, kSNP uses the jellyfish software [28], then compares these mers in all the genomes to find SNPs and finally creatse a SNP matrix to make phylogenetic trees. ParSNP uses maximal unique matches (MUM) to generate a multiple genome alignment and a SNP tree. This MUM for two genomes is a genetic index that considers the level of DNA conservation of the core genome and the proportion of DNA shared by these genomes. After the alignment process, the core-genome SNPs are selected and used to construct a phylogenetic tree.

### Cog analysis

To determine the most variable and conserved COGs for MTB, a COG analysis was performed on 249 MTB genomes that included the genotypes Beijing, CAS, EAI, Haarlem, LAM, X, Ural, AFRI1, AFRI2 and unknowns. Briefly, a comparative analysis based on H37Rv to determine that SNPs was performed using SamTools program [24]. Then, a SNP database was generated which included SNPs corresponding gene and COG.

### SNP set selection

Genotyping assignment was performed in two steps: (I) using the inclusion/exclusion criteria in experimental genotype (Spoligotypes) and phylogenetic prediction and, (II) using COG.

### (I) Inclusion/ exclusion (IE) criteria using spoligotyping and phylogenies

First, 125 MTB genomes with experimental information of genotypes were processed. Each genome sequence was transformed to binary codes, as presence (1) or absence (0) of a mutation in loci, based on H37Rv. Then, genomes were grouped according to their genotypes (Beijing, CAS, EAI, Haarlem, LAM, X, AFRI1 and AFRI2). Using IE criteria, loci shared with more than one genotype were discarded. Second, to reinforce the SNP selection, 124 additional MTB genomes (76 with genotypes determined by phylogenetic analysis and 48 of unknown genotypes with genotypes predicted by phylogenetic analysis) were included. Finally, the SNPs shared in more than one MTB genotype of Beijing, CAS, EAI, Haarlem, LAM, X, AFRI1 and AFRI2 were discarded. A total of 2123 specific SNPs to each genotype of MTB were identified as SNPs-IE.

### (II) Nonsynonymous SNPs selection based on COG (cluster of orthologous groups)

Then, the SNPs were organized in COG functional groups [29]. The genotypes of the MTB genomes have shown variant locus distribution in COG starting from the most variable to the most conserved (S, R, I, Q, E, C, F, L, N, G, H, K, P, J, T, O, F, D, V, U and A). In this case, we selected the locus from the most conserved to the most variable gene family. Then, we selected a locus associated with a nonsynonymous SNP for each of the 10 MTB genotypes.

## Results and Discussion

### Lineage determination of 48 unknown MTB genomes by phylogenic analysis

**Figure 1:**
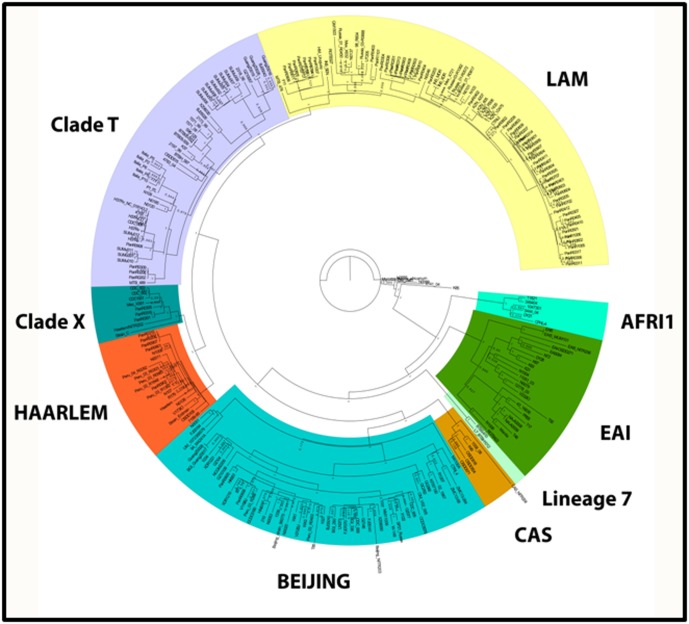
Phylogenetic tree under Maximum parsimony of 249 strains of M. tuberculosis.

Tree topology was performed on 249 MTB genomes using kSNP and Parsnp (Figure 1). Eight clades were identified with good support: AFRI1, EAI, CAS, Beijing, Haarlem, X, LAM and T. However, clade AFRI2 remains paraphyletic with strains at different levels of the bifurcation. Of the 48 unknown MTB genomes, 03 were AFRI1, 16 were EAI, 03 were CAS, 22 were Beijing, 15 were Haarlem, 03 were X and 15 were LAM. One Haarlem strain (Haarlem/NITR2) as determined by spoligotyping fell in the X clade by both methodologies (kSNP and Parsnp). There are limitations in the assignment of genotypes by phylogenetic analysis and often require global information of the genomic sequences for an optimal lineage approximation.

### Identification and selection of SNP set

**Figure 2:**
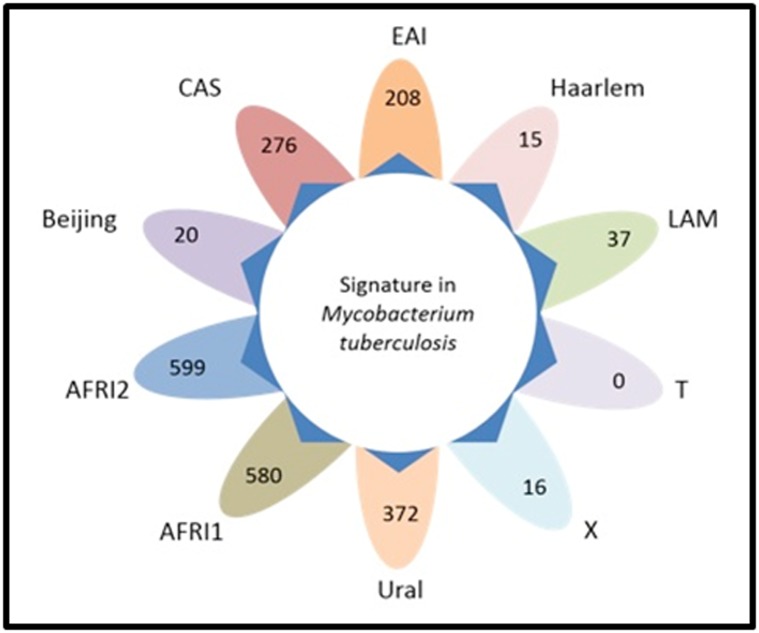
Diagram of signature SNPs for MTB genotypes: Beijing, CAS, EAI, Haarlem, LAM, T, X, Ural, AFRI1 and AFRI2 after IE criteria.

To develop the assigning system of SNPs and genotypes, we first used the IE criteria. 125 MTB genomes with known genotypes determined by spoligotyping were analyzed as described in Methods. The T genotype was not analyzed because the H37Rv falls in this genotype and was used in the process of genomic mapping. Additionally, 124 MTB genomes were included with genotypes determined by phylogenetic analysis. In a database, we integrated the 7649 SNPs from 249 genomic sequences of MTB that included the genotypes Beijing, CAS, EAI, Haarlem, LAM, X, Ural, AFRI1 and AFRI2. The number of non-specific genotype SNPs were 458, 854, 674, 624, 424, 412, 886, 1735 and 1582 respectively. After applying the IE criteria, the SNPs were organized in Group A, which contained SNPs unique for each genotype, and in Group B, which contained the SNPs shared between the different genotypes. Then, it was followed by a manual exclusion of shared SNPs. The result was a new group of SNPs (SNPs-IE) for the genotypes Beijing (n=20), CAS (n=276), EAI (n=208), Haarlem (n=15), LAM (n=37), X (n=16), Ural (n=372), AFRI1 (n=580) and AFRI2 (n=599) (Figure 2).

**Figure 3:**
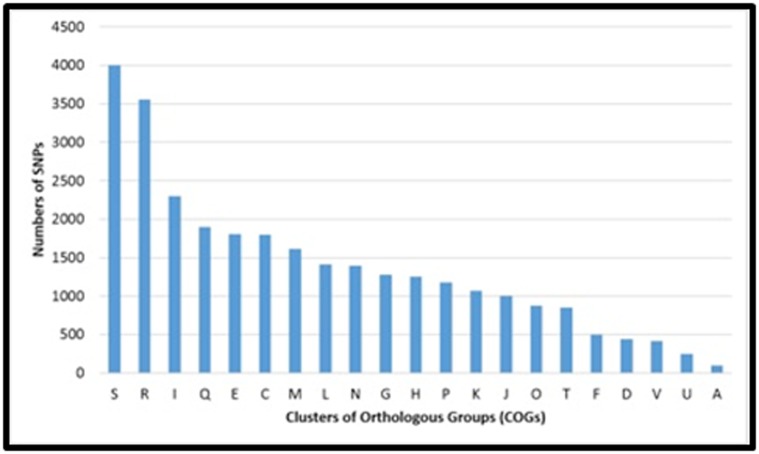
The relative distribution of SNPs of Mycobacterium tuberculosis in protein belonged to certain Clusters of Orthologous Groups (COGs). Function unknown (S), General function prediction only (R), Lipid transport and metabolism (I), Secondary metabolites biosynthesis, transport, and catabolism (Q), Amino acid transport and metabolism (E), Energy production and conversion (C), Cell wall/membrane/envelope biogenesis (M), Replication, recombination and repair (L), Cell motility (N), Carbohydrate transport and metabolism (G), Coenzyme transport and metabolism (H), Inorganic ion transport and metabolism (P), Transcription (K), Translation, ribosomal structure and biogenesis (J), Post-translational modification, protein turnover, and chaperones (O), Signal transduction mechanisms (T), Nucleotide transport and metabolism (F), Cell cycle control, cell division, chromosome partitioning (D), Defense mechanisms (V), Intracellular trafficking, secretion, and vesicular transport (U), RNA processing and modification (A).

Finally, this was followed by the selection of the nonsynonymous SNPs present in the most conserved COG of each of MTB. Before the analysis, the COGs most variables and most conserved for each genotype of MTB were determined (Figure 3). Then, a SNP database was generated, which included SNPs, corresponding gene and COG, showing 2, 36, 20, 4, 2, 2, 40, 65 and 67 SNPs for Beijing, CAS, EAI, Haarlem, LAM, X, Ural, AFRI1 and AFRI2 respectively. We selected 10 nonsynonymous SNPs for the 9 MTB genotypes analyzed (Table 1 and 2) and an alternative SNPs set (Table 2).

### Validating genotype assignment using new proposed set of 10 SNPs

Using an additional 34 MTB genomes with known genotypes Beijing (n=12), LAM (n=07), X (n=06), CAS (n=04), Haarlem (n=04) and EAI (n=01) we tested the ability of our new set of 10 SNPs to assign genotypes. There was 100% correlation of genotypes assignment with all the strains tested (Table 3).

## Conclusion

In conclusion, using the whole genome sequences of 249 MTB isolates, we identified a panel of 10 signature SNPs selected by combining IE criteria using spoligotyping and phylogenetic analysis and the selection of the nonsynonymous SNPs present in the most conserved cluster of orthologous groups (COG). This new proposed set of 10 SNPs can be used to build the molecular epidemiology of MTB, which can be incorporated in diagnostic assays and genotype-phenotype associations. The proposed system has the flexibility to be associated with the global databases where genotypes are based on the gold standard method: Spoligotyping. With the increase of genomes uploaded in global databases, future studies will obtain better support in the assignment of signature SNPs. The addition of the genomic signature along with drug resistance markers will improve TB control.

## Author Contribution

Conceived and designed the experiments: DT, HG. Performed the experiments: DT, LJ, VB. Analyzed the data: DT, LJ, VB, KL. Contributed materials/analysis tools: DT, LJ, HG. Wrote the paper: DT, LJ, VB, KL, MG, HG. Research as a Master's thesis: LJ. All authors have approved the final manuscript. All authors read and approved the final manuscript.

## Conflict of Interest

The authors declare no conflicts of interest.

## Figures and Tables

**Table 1:** Stepwise SNP set selection: IE, differential genotype ; *, the loci belonging to other strains from different genotypes and uncommon between genotypes were eliminated. **, SNP set based in COG group (AàK); *** SNP set based in less variable COG group genotype.

STEPS		Genotype	Beijing	CAS	EAI	Haarlem	LAM	X	Ural	AFRI1	AFRI2	T	Total
Gagneux [30]	L2	L3	L1 L3	L4	L4	L4	L4	L5	L6	L4	
MTB genome sequences	52	6	23	19	78	9	1	6	5	50	249
1	Integration of 7649 SNPs	Total SNPs	458	854	674	624	424	412	886	1735	1582		7649
2	IE criteria selection	SNPs-IE	69	310	220	33	93	56	396	737	621		2535
		SNPs - IE*	20	276	208	15	37	16	372	580	599		2123
3	Nonsynonymous SNPs selection present in most conserved COG	SNP set based COG group**	3	29	22	1	3	1	36	68	79		243
		COG in MTB most conservative	F	U	U	F	D	K	A	A	A		-
Proposed SNP set ***			1	1	1	1	1	1	1	1	2		10

**Table 2:** Set of 10 SNPs proposed to genotype MTB.

Genotype Differential		COG	Gene (locus tag)	SNP proposed
Principal SNPs set	Beijing	Nucleotide transport and metabolism	GuaB2 (Rv3411c)	3830349 (Ala391Thr: GCG ->ACG)
	CAS	Intracellular trafficking, secretion, and vesicular transport	(Rv3921c)	4409954 (Ala39Gly:GCC-> GGC)
	EAI	Intracellular trafficking, secretion, and vesicular transport	SecE1 (Rv0638)	734116 (Met127Thr: ATG->ACG)
	Haarlem	Nucleotide transport and metabolism	Hpt (Rv3625c)	4063682 (Leu61Met: CTG->ATG)
	LAM	Cell cycle control, cell division, chromosome partitioning	Smc (Rv2922c)	3236230 (Arg526Leu:CGT->CTT)
	X	Transcription	(Rv2618)	2946570 (Gly63Asp:GGC->GAC)
			(Rv3625c)	4063682 (Leu61Met:CTG->ATG)
	Ural	RNA processing and modification	(Rv1097c)	1225462 (Asp228Gly:GAC->GGC)
	AFRI1	RNA processing and modification	(Rv3439c)	3858894 (Leu266Phe:CTT->TTT)
	AFRI2	RNA processing and modification	(Rv1097)	1226021 (Gln42Lys:CAG->AAG)
			(Rv3689)	4130604 (Ser42Asn:AGC->AAC)
Alternative SNPs set	Beijing	Post-translational modification, protein turnover, and chaperones	(Rv1463)	1651308 (Glu198Gly: GAA ->GGA)
	CAS	Defense mechanisms	IrtA (Rv1348)	1513189 (Ala48Val: GCT-> GTT)
	EAI	Defense mechanisms	(Rv1730)	1956930 (Thr39Pro: ACT->CCT)
	Haarlem	Transcription	RpoC (Rv0668)	765150 (Gly594Glu: GGG -> GAG)
	LAM	Signal transduction mechanisms	CstA (Rv3063)	3429202 (Tyr654Asp: TAC -> GAC)
	X	Carbohydrate transport and metabolism	(Rv2994)	3352244 (Thr312Ala: ACC->GCC)
	Ural	Intracellular trafficking, secretion, and vesicular transport	FtsY (Rv2921)	3233940 (Ala67Gly: GCC->GGC)
	AFRI1	Intracellular trafficking, secretion, and vesicular transport	(Rv1887)	2136642 (Leu129Phe:CTT->TTT)
	AFRI2	Defense mechanisms	IrtA (Rv1348)	1515003 (Ala653Thr: GCC>ACC)

**Table 3:** Genotype assignment of 34 MTB genomes using new proposed set of 10 SNPs.

	Strain	Accesion Number	Ref. Lineage	Genotype assigned by our new set of 10 SNPs
1	13-2459	LDNL00000000	Beijing	Beijing
2	5351	JXXH01000000	Beijing	Beijing
3	96075	CP009426	Beijing	Beijing
4	B9741	LVJJ01000000	Beijing	Beijing
5	BEIJING-L 323	CP010873	Beijing	Beijing
6	BeijingDS 6701	JOKR01000001	Beijing	Beijing
7	E186hv	JXAW00000000	Beijing	Beijing
8	KT-0133	JUFG00000000	Beijing	Beijing
9	MTBR209	LATO00000000	Beijing	Beijing
10	W06	LHCK00000000	Beijing	Beijing
11	ZT272	LGTJ00000000	Beijing	Beijing
12	TBR-103XDR	JRJT01000001	Beijing	Beijing
13	tahitMT11	CVMX01000001	Haarlem	Haarlem
14	TBR-102	JRJS00000000	Haarlem	Haarlem
15	TKK_03_0101	GCF_000651975.1	Haarlem	Haarlem
16	TKK_03_0103	GCF_000651995.1	Haarlem	Haarlem
17	TBR-152	JRJQ00000000	LAM	LAM
18	TKK_04_0029	GCF_000673435.1	LAM3	LAM
19	TKK_04_0038	GCF_000673275.1	LAM4	LAM
20	TKK_04_0039	GCF_000673295.1	LAM4	LAM
21	TKK_04_0043	GCF_000673075.1	LAM4	LAM
22	TKK_04_0044	GCF_000673335.1	LAM3	LAM
23	TBR-175	JRJR00000000.1	LAM	LAM
24	TKK_04_0120	GCF_000654175.1	EAI	EAI
25	TKK-01-0028	GCF_000660665.1	X	X
26	TKK_02_0027	GCF_000672095.1	X	X
27	TKK_03_0063	GCF_000651695.1	X	X
28	TKK_03_0099	GCF_000651935.1	X	X
29	TKK_03_0150	GCF_000652255.1	X	X
30	TKK_05SA_0021	GCF_000653515.1	X	X
31	TKK_03_0037	GCF_000651475.1	CAS	CAS
32	TKK_04_0139	GCF_000656875.1	CAS	CAS
33	TKK_04_0148	GCF_000656935.1	CAS	CAS
34	TKK_05SA_0050	GCF_000653755.1	CAS	CAS
